# Inventory management performance for laboratory commodities and their challenges in public health facilities of Gambella Regional State, Ethiopia: A mixed cross-sectional study

**DOI:** 10.1016/j.heliyon.2022.e11357

**Published:** 2022-11-02

**Authors:** Bekele Boche, Shamoro Temam, Oliyad Kebede

**Affiliations:** a Department of Social and Administrative Pharmacy, School of Pharmacy, Jimma University, Jimma, Oromia, Ethiopia; b Gembella Regional Health Bureau, Gambella, Ethiopia; c Department of Social Pharmacy and Pharmaceutics, School of Pharmacy, College of Medicine and Health Sciences, Mizan-Tepi University, Mizan-Aman, Ethiopia

**Keywords:** Ethiopia, Gambella regional state, Health facilities, Inventory management, Laboratory commodities

## Abstract

**Background:**

Sustaining an adequate inventory level of laboratory commodities is critical as 70% of medical decisions are made using laboratory-related information. However, millions of populations in developing countries still do not get elementary laboratory services due to the inaccessibility of laboratory commodities. Hence, this study aimed to assess inventory management performance and challenges for laboratory commodities in public health facilities of Gambella regional state, Ethiopia.

**Method:**

A health facility-based descriptive cross-sectional study was conducted using a mixed quantitative and qualitative method in seventeen public health facilities of the Gambella regional state between May and July 2021. Data were collected from documents, health professionals, and health care facilities' warehouses. Quantitative data were analyzed using Excel spreadsheets version 14 and SPSS version 23. In the qualitative part, 18 key informants were interviewed, and data were analyzed using thematic analysis techniques.

**Result:**

The average availability of laboratory commodities on the day of the visit was 60.39% in health facilities. Over the past year, 12.94% (37,488.76 US$) of laboratory commodities were wasted due to damage and expiration, while the average stock out date was 58 days. The average accuracy, completeness, and timeliness of the report and requisition form were 49%, 71%, and 64%, respectively. The health facilities had attained only, 68.2% of the storage conditions criteria. Lack of adequate and committed staff, budget constraints, insufficient storage space, frequent shortages of commodities from the suppliers, lack of frequent supervision, and feedback from higher officials were the main challenges for inventory management of laboratory commodities.

**Conclusion:**

The inventory management for laboratory commodities was inadequate, which was disclosed by inaccurate records and reports, stock-outs, high wastage rate, lack of adequate staff, budget constraints, and unacceptable storage conditions criteria. The study result suggests strict and close monitoring is needed to improve the inventory management performance for laboratory commodities at health facilities.

## 1 Background

Medical laboratory services are a critical part of the health care system for appropriate diagnosis, management, and prevention of communicable and non-communicable diseases. To provide these services, laboratory commodities are required, which are used to collect, prepare, test, analyze, store and dispose of clinical specimens to deliver laboratory service for patients at health facilities. These products are basic items to forward the necessary results required for further decisions of clinicians on the selection of appropriate treatment options in disease control, prevention, and investigation. About 70% of medical decisions are made using laboratory related information in health facilities. Such provision of quality laboratory services requires reliable access to laboratories commodities [1, 2, 3, 4].

Proper logistic management for laboratory commodities expedites uninterrupted accessibility of products, wise use of available resources; justifiable need anticipating, inventory management, information management, and customer relationship management [5, 6]*.* Inventory management is a core operational component of supply chain management that provides accurate, complete and timely information for the storekeeper when to order or issue, what proportion to order or issue, way to sustain an acceptable stock level of all products to avoid shortages and oversupply. Moreover, after laboratory commodities are procured and received by health facilities, they should be maintained under appropriate storage conditions. Retaining an acceptable level of inventory is critical as a huge amount of capital is tied up with it. Having excess inventory leads to wastage of products through expiration and damage, while too little inventory leads to stock-outs that lessened quality of patient care [7, 8, 9]. Availability of organized quality information in inventory management of laboratory commodities facilitate product availability, promote the continuation of community services, increase the quality of care, and improve job satisfaction and morale of healthcare professionals. A motivated staff is more likely to provide better service and ensure efficient health delivery [10].

Additionally, laboratory commodities inventory management supports store keepers for easily monitoring expiration time, stock levels, impenetrable losses, and storage situations, lesser procurement assignments, and it is particularly serious for test kits and investigative reagents which have a little shelf life [11].

Laboratory services are an essential part of the healthcare system, however, inventory management of laboratory commodities used for these services are challenging because of most laboratory products have a short shelf life, require special storage conditions, the single test requires different products, and laboratory products can be packaged in bulky items such as kits, consumable liquids, and dry laboratory chemicals [12, 13]. This challenge is pressing in middle and low-income countries due to their giving less attention to laboratory services as part of the health care system, a lack of resources, inadequate logistic activities, and poor administrative support. Consequently, millions of populations in these countries still do not have access to reliable and elementary laboratory services [14, 15]. For instance, studies conducted in Ghana, Malawi, Zimbabwe, Rwanda, Tanzania, and Ethiopia revealed that inappropriate laboratory commodities inventory management due to limited human resources, lack of appropriate skill, insufficient funds, lack of quality data at the health facilities level, weak information technology, and insufficient storage space for products. All these cause inadequate product availabilities, inaccurate diagnosis and laboratory reports, service interruptions, and patient dissatisfaction [16, 17, 18, 19, 20, 21].

The Ethiopian Federal ministry of health and Pharmaceuticals supply agency primarily focuses on and struggles to ensure that all citizens have access to quality medical laboratory services. However, the country's laboratory management remained weak, redundantly being troubled by the lack of quality information for procurement and resupply decisions, an insufficient supply of required commodities, and wastage of items due to overstocking, expiry, and damage. All these bottlenecks caused recurrent stock-outs of critical products, thus hampering uninterrupted and quality testing for patients [10, 21]. For example, Methanol, Mask, Carbol fuschin, methyl blue, pregnancy test, HIV screening test kit, and HIV confirmatory test kit were stocked-out on average for 51.2, 54.7, 17.7, 21.6, 16.3, 31.9, and 44.1 days, respectively [3]. While, Gram stain reagent iodine, Gram stain reagent alcohol, Acid-alcohol solution, Hematology auto-analyzer reagent, and Chemistry auto-analyzer reagent kit wastage rate was 78.6%, 63.9%, 60%, 36.6%, and 34.3% respectively from its annual budget [2].

Despite a few studies mentioned above, which reveal inadequate laboratory commodities inventory management and many challenges in Ethiopia, there are no studies conducted in the Gambella region, southwestern Ethiopia. Gambella region is one of the remotest and located in the lowlands of Ethiopia bordering with the Republic of South Sudan where high attrition rate of health professionals and the difficult weather conditions which challenges laboratory products management, and poor health system infrastructures [22]. Therefore, this study aimed to assess inventory management performance for laboratory commodities and its challenges in public health facilities of Gambella Regional State, Ethiopia.

## 2 Method

### 2.1 Study setting, design and period

The study was conducted in public health facilities of Gambella regional state, southwest Ethiopia. Gambella regional state is one of the ten member states of the Federal Democratic Republic of Ethiopia, with an area of 29,783 km^2^ and located 777 km away from the capital city of the country, Addis Ababa. The region has three zones; with 14 woredas and 165 kebeles. In Gambella region, there were 175 public health institutions, including health posts (n = 142), health centers (n = 28), and public hospitals (1 general hospital, 4 primary hospitals) during the study period. A health facility-based descriptive cross-sectional study was conducted using a mixed quantitative and qualitative method in 17 public health facilities of the Gambella region between May and July, 2021.

#### 2.1.1 Source populations, study units, and data sources

The source populations for this study were all public health facilities in Gambella regional state, all laboratory commodities in those facilities, all health professionals, and all logistics documents used to manage laboratory commodities in these facilities. In Gambella regional state, there were 33 public health facilities (five hospitals and 28 health centers). Since, they do not manage majority of laboratory commodities, health posts were not included in this study. Therefore, the study units for this study were selected health centers and hospitals, health professionals entitled to manage laboratory commodities in those selected facilities, and logistics documents used to manage laboratory commodities. The data sources were pharmacy and laboratory professionals, logistics documents (bin-cards, report and requisition format (RRF), Health Commodity Management Information System (HCMIS), archived documents of wasted items due to expiration and damage, and transaction document (model 19 and model 22)).

#### 2.1.2 Eligibility criteria

Public health facilities (public hospitals and Health centers), pharmacy and laboratory professionals who served in managing laboratory commodities for more than six months in selected facilities, and all laboratory commodities were eligible for this study.

### 2.2 Sampling size determination and sampling procedures

#### 2.2.1 Sample size for health facilities

Since, their number was manageable for the this study, we included all hospitals in the region (N = 5) and we determined the sample size for health centers using Logistic Indicators Assessment Tool (LIAT) developed by USAID/DELIVER PROJECT, which recommend a sample size 15% of the targeted health facilities [23]. Accordingly, the sample size for health centers became:

n = N × 15% = 28 ∗ 15% = 5 health centers

where N is the total number of health centers and n is the sample size for the health centers.

However, the region has administratively 14 woredas, which could not represented by only 5 health centers. Therefore, we took 1 health center from each woreda which increased the sample size for health centers to 12. Two of the woredas had no health center. Each health center was selected from each woreda using lottery method. Finally, 12 health centers (1 from each woreda) and five hospitals were included in the study.

#### 2.2.2 Sample size for laboratory commodities

Regarding laboratory commodities, we included the core laboratory items listed in Assessment Tool for Laboratory Services (ATLAS) [24], which are 28 core laboratory commodities in total (supplementary file one).

#### 2.2.3 Sample size for documents

Each laboratory commodity should have 1 bin card in each facility. Therefore, 28 bin-cards were anticipated from each health center and hospital, making 476 (28 ∗ 17) bin cards from 17 health facilities. In addition, there were six RRFs expected in one year from each health facility, because each facility reports and requests laboratory commodities every two months. Totally, 102 RRFs were reviewed. Health Commodity Management Information System (HCMIS)/Dagu facility, archived documents of expired or damaged items, and receiving voucher (model 19) were reviewed. These documents were used to determine the LMIS utilization, availability and wastage rate of laboratory commodities. For quantitative data, we administered self-administered questionnaires to 17 store managers and 17 laboratory managers (2 individuals from each facility). They were selected purposively, because they are individuals primarily responsible for laboratory commodities management.

### 2.3 Data collection procedures

Structured questionnaires and checklists adopted based on the LIAT [23] and ATLAS [24] were used to collect the quantitative data. The data were obtained through document assessment using checklists, observation of commodities storage areas and, from store and laboratory managers using self-administered questionnaires. The structured questionnaire had two parts. The first part consisted of the socio-demography of store and laboratory managers. Moreover, the second part contained questions dealing with facility characteristics such as supervision from upper stream, ordering and receiving activities, and availability and utilization of LMIS tools. The checklists were used to collect data to determine availabilities, stock out, wastage rates, bincard updating practices, RRF accuracy and storage conditions of laboratories stores. We have recruited 5 data collectors (3 pharmacists and two medical laboratory professionals), who were familiar with the study area. The principal investigators gave one day training for data collectors on the study objective and data collection procedure.

### 2.4 Data processing and analysis

Data were entered into Epidata version 3 for cleaning and exported to SPSS version 23 for analysis. The Excel spreadsheet version 14 was also used to calculate stock out, and wastage rates. Then, the results were transmitted to SPSS for more analysis. The results were then presented using charts, graphs, and texts. The measurement formula used for quantitative data was described in detail (supplementary file two).

### 2.5 For the qualitative study

In-depth face-to-face interviews were conducted with eighteen key informants (KIs). The number of KIs was dependent on information saturation, i.e., the interviews terminated as soon as the new interviewee repeated what had already been said. Moreover, the selection of the participants took into consideration the service year and the role in the laboratory commodities logistics management. Exploratory and penetrating questions were adopted from the logistics system assessment tool (LSAT) [25] to explore the challenges for inventory management of laboratory commodities. The interviews were performed physically face-to-face with key informants, and the principal investigator performed the conversation to guarantee data consistency. The interviews were conducted in local languages, Amharic, and the records were audio typed and notes were taken. On average, each interview lasted for 25 min. The interviews were held at each respondent's workplace, and mutual sympathetic was established with each key informant. Regarding participant consent and confidentiality, written informed consent was received from each respondent ahead of the interview according to Helsinki principles. The principal investigator informed key informants ahead of starting the discussion; that participating and not participating in the study is their full right, and they can stop participating in the study at any time. The study has no risk for the participants as the name of the key informant is not described in the report; and audio recorded type is kept private by investigators during transcription, translation, and report writing, and their confidentiality was retained private. After going over it, the investigators transcribed the recording into the English language. Then, one of the qualitative research specialists at Jimma University proved the accuracy of the transcription.

The analysis was performed manually using a thematic technique. Codes have been given to variables and then drawn together in a tabular format. Variables with a similar code were organized to formulate appropriate themes. These include problems related to health facilities and suppliers, human resources, availability of commodities, LMIS, Supervision, and monitoring. We then identified the themes and quoted the views of the interviewees to explain the seriousness of the issues.

### 2.6 Data quality assurance

We conducted a pretest on 5% of the sample size in health facilities (2 health center) with similar service and setting found in the region. Health centers selected for pre-test were not part of the study sample. The pretest was done to approve the understandability and accuracy of the tools. During the data collection process, the principal investigators supervised the data collectors frequently to ensure data completeness. To maintain consistency throughout the interviews, the qualitative data were collected by principal investigators. The qualitative data report were brought to all investigators and discussed before final report. Then, all investigators gave comments and final report was produced.

#### 2.6.1 Ethics approval and consent to participate

Ethical clearance has been obtained from the Ethical Review Board of Jimma University, institute of health with the reference number of IHRPGD/185/2021 and issued on 17/5/2021. Then official support letters from the post-graduate program coordinator and pharmacy school were submitted to Gambella regional health bureau (GRHB) then after a letter of permission was obtained from GRHB, and a letter was written to the respective hospitals and health centers. During the study, professional and social ethics have been maintained and the name of the facilities and personnel involved in the study has not been stated on the result analysis thus the confidentiality of the information was assured.

### 2.7 Operational definitions and definition of terms

#### 2.7.1 Availability

Laboratory commodity that is available in the storeroom, which is equal to or above average daily consumption on the day of visit [26].

#### 2.7.2 Bin-card accuracy

Is a gauge of a divergence between the stock balance on bin-cards and physical inventory balance of laboratory commodities.

#### 2.7.3 Bin card update

The bin card is updated if it had to be updated within the previous 30 days unless last updated with the balance of zero or the facility has not received any of those products [2].

#### 2.7.4 Completeness

A report is measured as complete if all the columns for each laboratory commodity recorded in the report are filled in for at least one item listed below each program.

#### 2.7.5 Data quality

It is the accuracy, completeness, and timeliness of Laboratory commodities data.

#### 2.7.6 RRFs accuracy

A measure of inconsistency between the Laboratory commodities balance on RRFs and the ending balance on bin-cards. RRF is said to be accurate if the record balance difference between RRFs and ending balance on bin cards is zero [2].

#### 2.7.7 Health facilities

Health centers and hospitals.

#### 2.7.8 Kebele

Sub-districts/woredas, and the smallest level of administration structures in Ethiopia.

#### 2.7.9 Laboratory commodities

Include key supplies, chemicals, reagents, and kits used for running laboratory tests in the study settings. It used exchangeable with products/items.

#### 2.7.10 Waste

Unused laboratory commodities because of expiration or damage.

#### 2.7.11 Woreda

It is used interchangeably with the district, and refers to the third level of the administration of Ethiopia, after region and zone.

## 3 Result

### 3.1 Characteristics of respondents

Laboratory heads and medical store managers were principal personnel for laboratory commodity supply management in assessed facilities. The socio-demographic characteristics of medical store managers and laboratory heads showed that, the profession of majority of the laboratory heads 11 (64.7%) were laboratory technicians and majority 13 (76.5%) of store managers took integrated pharmaceutical logistic system (IPLS) training. Moreover, 15 (88.2%) of the store managers had a work experience below 5 years (Table 1).

**Table 1:** Socio-demographic characteristics of respondents in selected public health facilities of Gambella regional state, January 2022.

Profession	Qualification	Years of experience		Training received		
		<5	5–10	LCM^a^	IPLS^a^	Total
**Store Managers**						
Nurse	Diploma	5 (29.4%)	0	1 (5.9)	4 (23.5%)	5 (29.4)
	Degree	0	0	0	0	0
Pharmacy	Diploma	8 (47.1%)	0	3 (17.6%)	5 (29.4%)	8 (47.1)
	Degree	2 (11.8%)	2 (11.8%)	0 (0%)	4 (23.5%)	4 (23.5%)
**Laboratory Head**						
Medical laboratory	Diploma	11 (64.7%		0	0	
	Degree	6 (35.3%)		3 (17.6%)	0	

LCM^a^ = laboratory commodity management. IPLS^a^ = integrated pharmaceutical logistics system.

### 3.2 Inventory management of laboratory commodities

Ten (58.82%) of the assessed health facilities were following the established minimum & maximum inventory control levels for laboratory commodities. However, all of them have been conducted annual inventory physical verification of LC. Pharmacy units alone were responsible for order placing in three hospitals and nine health centers. Regarding the resupplying of LCs for health facilities, Gambella hub supplied 13 (76.5%) of selected health facilities, whereas the rest were supplied by Jimma hub. None of the assessed health facilities received all orders they requested from the suppliers, and all of them had a history of placing an emergency order (EO) at least once in the last year. Consequently, an overall average of 7.29 emergency orders per year was placed from each health facility. The minimum resupply lead-time for the order of laboratory commodities was 14 days, and on average, 33.3 days for assessed health facilities (Table 2).

**Table 2:** Laboratory commodities’ inventory management practice in public health facilities of Gambella regional state, January, 2021.

Inventory management practice of laboratory commodities					
Variables			Hospitals		Health centers
			Frequency (%)		Frequency (%)
Conduct an annual physical count for LCs	Yes		5 (100%)		12 (100%)
	No		0 (0%)		0 (0%)
Supplying EPSA	Gambella hub		4 (80%)		9 (75%)
	Jimma hub		1 (20%)		3 (25%)
Follow established Min/max, and Reorder levels for LCs	Yes		5 (100%)		5 (41.7%)
	No		0 (0%)		7 (58.8%)
Responsible body to determine order	Pharmacy unit		3 (60%)		9 (75%)
	Pharmacy & laboratory unit		2 (40%)		3 (25%)
Received all times full quantity requested	Yes		0 (0%)		0 (0%)
	No		5 (100%)		12 (100%)
	Minimum	Maximum	Range	Average	Standard Deviation
EO placed last year	1	12	11	7.31	3.088
Lead time orders (day)	14	60	46	33.3	13
Health facility distance (km) from supplying EPSA	0.5	491	490.5	158.8	140.7

LC = laboratory commodities, Km = Kilometer, EPSA; Ethiopia pharmaceutical supply agency.

### 3.3 Availability of laboratory commodities in public health facilities

On average, the availability of assessed LC on the day of the visit was 65.71% in hospitals and 55.06% in health centers. The average stock out duration of LCs in hospitals and health centers was 19.86 and 96.32 days, respectively in the last year. Likewise, among those selected laboratory commodities, hematology reagent, Chemistry reagent kits of GOT (AST), Glucose, and Creatine have not been available in any health centers on the day of visit. Whereas, HIV 1st test kit (stat pack), HIV 2nd test kit (Abon), immersion oil, and pregnancy test kit were the most common available reagents on the day of visit in selected public health facilities (Supplementary file three).

### 3.4 Wastage of LCs

Regarding the wastage of LC due to damage and expiration in last year, 16 of the selected laboratory commodities had a history of wastage with an average rate of 12.94%. Acid alcohol and carbol fuchsine account for 37.5% and 31.3%, respectively. In terms of value, 1,186,353.3 (37,488.76 US$) Ethiopian Birr (ETB) was lost due to expiry and damage of laboratory commodities (Table 3).

**Table 3:** The wastage rate and value of laboratory commodities in public health facilities of Gambella regional state, January 2022.

S .N	List of LCS	Amount wasted	Amount available	Unit Price (US$)	Unit Price (ETB)	Total waste (ETB)	Wastage rate (%)
1	Acid alcohol	36	96	2.234	71.65	2579.4	37.5
2	Safranin	18	147	1.53	49.0	882	12.2
3	Carbol fuchsine	93	297	2.03	65.0	6045	31.3
4	HIV test kit 1st test	462	8031	13.46	431.54	199371.48	4.2
5	CD4 reagent	48	366	560.54	17966	862368	13.1
6	VDRL	75	663	18.25	585	43875	11.3
7	Blood group	15	294	8.34	267.2	4008	5.1
8	Hepatitis test kit	21	663	22.856	732.66	15385.86	3.2
9	Geimsa stain	21	432	1.75	56.0	1176	4.9
10	ALP/GOP	12	78	33.68	1079.5	12954	15.4
11	Ceratinie	9	99	28.92	927	8343	9.1
12	Immersion oil	42	387	2.33	74.77	3140.34	10.9
13	Urine multi test	66	483	4.73	151.75	10015.5	13.7
14	Grams Iodine	18	159	1.47	47.01	846.18	11.3
15	Crystal violet	6	111	3.06	98.0	588	5.4
16	HCG	114	672	4.04	129.61	14775.54	16.96
	Average					1,186,353.3 (37,488.76 US$)	12.94

LCs= laboratory commodities, ETB= Ethiopian Birr

### 3.5 LMIS implementation performance of laboratory commodities

**Figure 1:**
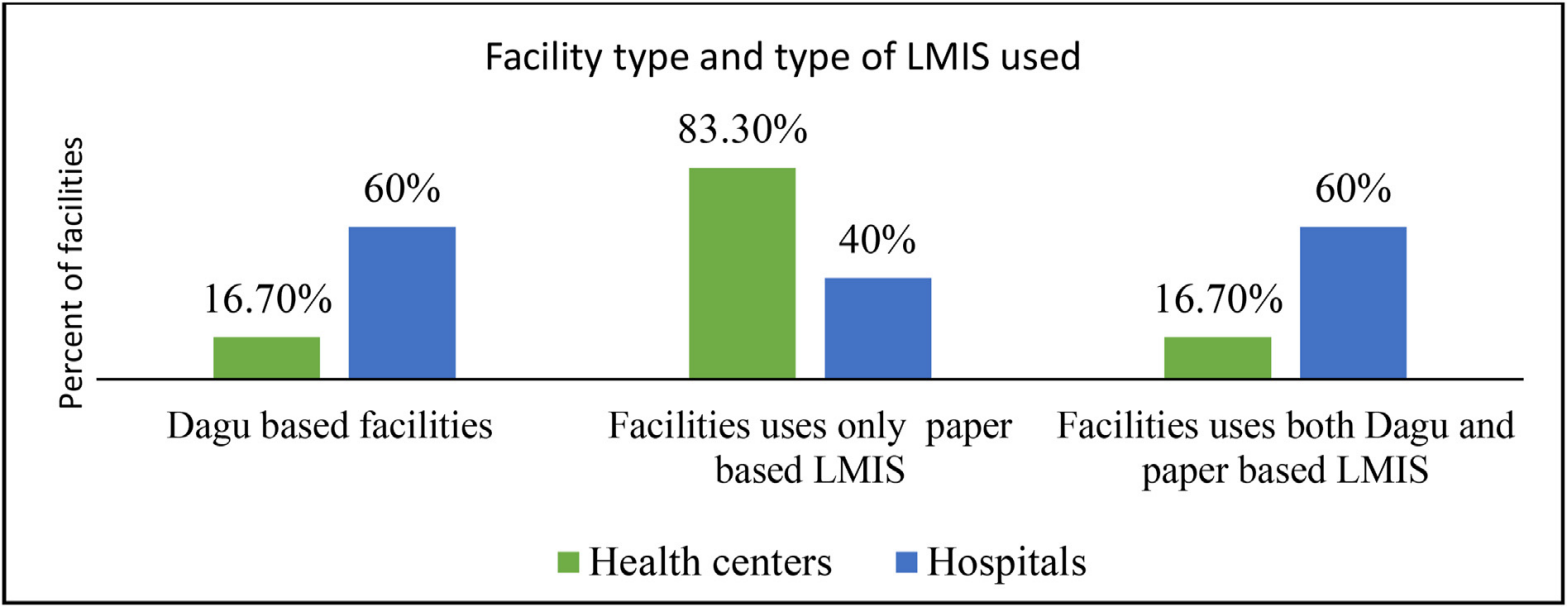
Types of LMIS used for laboratory commodities in public health facilities of Gambella regional state, January 2022. LMIS =logistics management information system, Dagu based= electronic LMIS.

There were manual and electronic information management systems in the assessed health facilities. Among them, the majority, 12 (83.3%), of health facilities used only manual (paper based) LMIS. But, three hospitals and two health centers were using both paper and electronic LMIS to control laboratory commodities (Figure 1).

From 476 bin cards expected in all assessed health facilities, only 280 (58.8%) were found. Out of that, 90 (64.3%) were at hospitals, while 190 (56.54%) were at health centers. However, only 50 (35.7%) and 115 (34.2%) of them were updated in hospitals and health centers, respectively. HIV testing kits and immersion oils had better available bin cards at most health facilities (Table 4).

**Table 4:** Bin cards availability and updating practice in selected public health facilities of Gambella regional state, January 2022.

S. No	Laboratory commodities	Frequency (%) Bincard available for LC		Frequency (%) Bincard updated	
		Hospitals	Health centers	Hospitals	Health centers
1	HIV test kit 1st response	4 (80%)	11 (91.7%)	4 (80%)	10 (83.3%)
2	HIV test kit 2nd response	5 (100%)	11 (91.7%)	3 (60%)	8 (66.7%)
3	HIV test kit 3rd response	4 (80%)	6 (50%)	1 (20%)	1 (8.3%)
4	Gram stain reagent, safranin	0 (0%)	5 (16.7%)	0 (0%)	2 (16.7%)
5	Gram stain reagent, iodine	1 (20%)	5 (16.7%)	0 (0%)	3 (25%)
6	Gram stain reagent, alcohol	4 (80%)	8 (66.7%)	3 (60%)	6 (50%)
7	Gram stain reagent, crystal violet	0 (0%)	3 (25%)	0 (0%)	3 (25%)
8	Acid-alcohol solution	5 (100%)	9 (75%)	3 (60%)	7 (58.3%)
9	Carbol Fuchsine 1%	4 (80%)	8 (66.7%)	2 (40%)	7 (58.3%)
10	Methylene blue solution	3 (60%)	8 (66.7%)	2 (40%)	5 (41.7%)
11	Methanol	3 (60%)	5 (41.7%))	3 (60%)	2 (16.7%)
12	Giemsa stain solution	3 (60%)	11 (91.7)	3 (60%)	8 (66.7%)
13	Potassium hydroxide 10%	2 (40%)	1 (8.3%)	1 (20%)	1 (8.3%)
14	Hematology auto analyzer reagent kit	4 (80%)	0 (0%)	1 (20%)	0 (0%)
15	Chemistry auto analyzer reagent kit, GOT (AST)	2 (40%)	0 (0%)	1 (20%)	0 (0%)
16	Chemistry auto analyzer reagent kit, glucose	5 (100%)	0 (0%)	0 (0%)	0 (0%)
17	Chemistry auto analyzer reagent kit, creatine	4 (80%)	0 (0%)	1 (20%)	0 (0%)
18	Blood group/type antisera	4 (80%)	6 (50%)	1 (20%)	1 (8.3%)
19	Immersion oil	5 (100%)	12 (100%)	5 (100%)	9 (75%)
20	Pregnancy test kit	4 (80%)	8 (66.7%)	4 (80%)	7 (58.3%)
21	Urine multi-test	3 (60%)	10 (83.3%)	2 (40%)	4 (33.3%)
22	Hepatitis screening	4 (80%)	9 (75%)	3 (60%)	4 (33.3%)
23	RPR/VDRAL kit	4 (80%)	10 (83.3%)	3 (60%)	2 (16.7%)
24	CD4 test reagents Kit	2 (40%)	4 (33.3%)	2 (40%)	3 (25%)
25	Alcohol 70%	2 (40%)	11 (91.7%)	1 (20%)	9 (75%)
26	Microscope slide	5 (100%)	9 (75%)	2 (40%)	6 (50%)
27	Glove	2 (40%)	9 (75%)	0 (0%)	4 (33.3%)
28	Mask	2 (40%)	11 (91.7%)	0 (0%)	3 (25%)
**Overall average**		**64.3%**	**56.54%**	**35.7%**	**34.2%**

LCs= laboratory commodities

**Figure 2:**
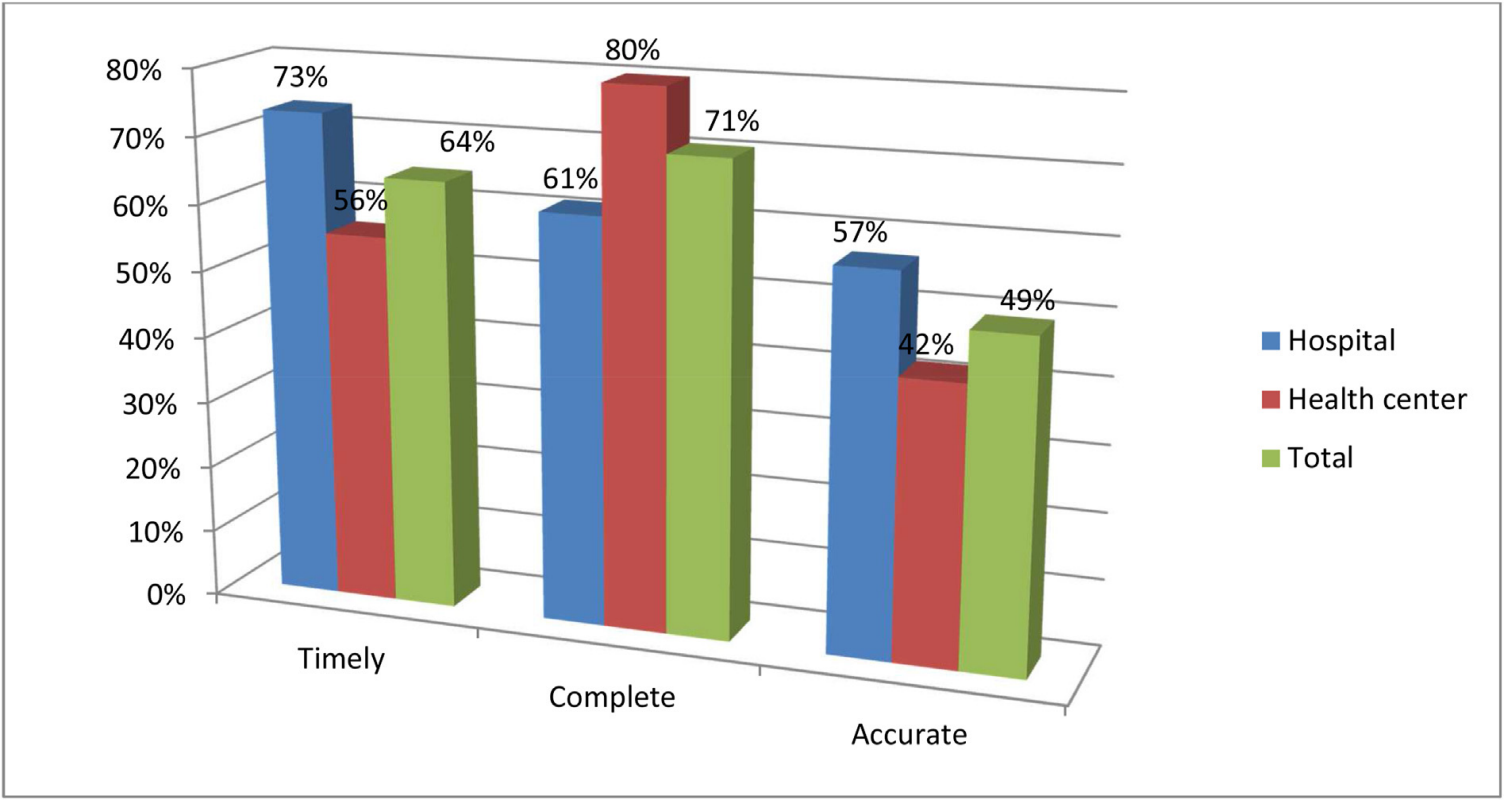
RRF timeliness, accuracy, and completeness practice in selected public health facilities of Gambella regional state, January 2022. EPSA = Ethiopian pharmaceutical supply agency, RRF = Report and Requisition form.

From 102 assessed RRF, only 49 % were accurate, 71% complete, and 64% timely reported to resupply facilities. The accuracy of RRF was 57% and 42% in hospitals and health centers, respectively (Figure 2).

### 3.6 Storage practice of laboratory commodities

According to the assessment, all of the health facilities complied with the storage area secured with a lock and key, as well access is limited to authorize personnel. In addition, the majority of them conceded through protecting their products from direct sunlight and maintaining their store in good condition & clean. However, in 14 (82.4%) of the facilities, there were not separating damaged and expired products from useable products and removing them from inventory and hadn't sufficient space and organization for accommodating existing products. Generally, the overall average performance of public health facilities that complied with the acceptable storage criteria was 68.2% (supplementary file four).

### 3.7 Inventory management challenges for laboratory commodities

#### 3.7.1 Health facilities and suppliers related challenges

Maintaining proper storage conditions reduces damage and simplifies tracing and tracking. However, in the current study setting, key informants reported there were no appropriate racks and shelves, insufficient storage space, less functional cold chain equipment, poor product arrangement, zoning, and FEFO principles not used for LCs. One key informant said that; “We have used one small room for both storing and managing all kinds of pharmaceuticals including laboratory commodities; in addition refrigerators not working continuously due to frequent power interruption. Therefore, it is difficult to apply FEFO, to separate useable stock from non-useable stock & even challenging to fulfill most of the acceptable storage requirements.”

In addition to health facilities-related challenges, another headache is the issue of pharmaceutical suppliers. Most of the key informants mentioned that there was redundant stock out, an undersupply of the laboratory commodities from EPSA, and supplier's long lead time was the major bottleneck for laboratory product availability.

### 3.8 Human resources related challenges

The key informants have elucidated the problems of skilled human resources as follows; lack of adequate staff, overload of activities, lack of training for all staff, high staffs ‘turnover rate, and lack of trained pharmacy professionals. One of the key informants described the challenge as follows; “*My profession is a nurse, and I am here to manage the store, which is not my right position, and for my duty, I am working in outpatient department as a nurse. I do not have an extra incentive for managing the store.” in addition, most health professionals are leaving the health facilities due to poor infrastructure, difficult weather conditions, and poor utility.”*

### 3.9 Challenges related to availability

As revealed by quantitative results stock-out of LCs was among the problems identified in assessed facilities. The causes of this problem were probed with KIs, and identified budget constraints to purchase products, frequent stock out from the supplier, closed system laboratory machines reagents provided by a single supplier, short shelf life due to the nature of LCs as contributing factors. For example, one of the store managers stated the problem as follows; “We put orders every two months for program laboratory commodities, but EPSA took more than a month to refill. As a result, we have been forced to request emergency orders between our reporting periods. In addition, due to budget insufficiency and even available budget is not released timely for us to purchase products. Regarding the availability of laboratory commodities for closed system laboratory machines, is frequent stock out of reagents from sole suppliers. I heard that there is initiative direction set by EPSA to solve the current problem, but still not started.”

### 3.10 LMIS related challenges

Another critical issue raised by KIs was LMIS related challenges. KPIs were raised different challenges for not keeping and reporting accurate, complete, and timely logistics information. Among these challenges were; lack of time due to workload, lack of electronic information management system, lack of awareness about the importance of quality data for drug supply management, lack of incentives for the extra workload.

A 33 years old men working as a pharmacy store mentioned as follows; ‘‘My profession is druggist, and I am here both to manage the store and the dispensary. In addition, I am busy with most recording and reporting formats that were not filled and updated regularly. Since the recording and reporting are done manually, it takes time. Again, there is no extra payment for doing such time taking work.”

### 3.11 Supervision and monitoring related challenges

Supervision and monitoring are the essential elements in drug supply management that help to improve the quality of the health services delivery. This may be through maintaining relationships between the staff and focusing on the identification of gaps and taking interventions to address the gaps, as well as motivating health professionals to boost their skills and performance. However, most key informants mentioned poor and infrequent supervision, lack of support and feedback.

A 40 years old man working as pharmacy store said; “My profession is a pharmacist, and I have worked in various health centers and hospitals, and currently, I have been the store manager at this hospital since last year. Lack of frequent supervision from the top responsible body such as the regional health bureau and EPSA. In addition, they did not support the development of staff skills and they give less attention for pharmaceutical services. As a result, patients often wake up and complain about the services provided by the pharmacy and laboratory services due to the frequent out stock of laboratory commodities.”

## 4 Discussion

Adequate inventory management of laboratory products plays an indispensable role in improving the accessibility of commodities, reducing inventory costs, and improving patient care services.

Even though laboratory commodities are an integral part of the health care system, their management is challenging because most laboratory items have short shelf lives, require special storage conditions, and single tests that require different products. Assimilation of these bottlenecks needs better LMIS management, close monitoring of stock levels and storage conditions, staff skills, and commitment [12, 26].

In the current study, the average availability of laboratory commodities on the day of the visit was 65.71% in hospitals and 55.06% in health centers. The average stock-out duration of LCs in hospitals and health centers was 19.86 and 96.32 days, respectively, and on average 58 days. It was a long period when compared with similar studies conducted in Jimma in 2019 (3) and 2020 (2), where the average stock-out duration was 51 and 8.5 days, respectively. The inconsistency in the result may be due to the study area. The current study is conducted in the Gambella region, the remotest area of the country with poor infrastructure, relative to the Jimma zone. Again, the previous study was conducted on hospitals for which the stock out duration is shorter than the current finding. However, there is a long duration of stock out in health centers in current findings than hospitals. From the key informants, the contributing factors for this problem were budget constraints, frequent stock-out from the supplier, closed system laboratory machines reagents provided by a single supplier, short shelf life due to the nature of LCs. Similar findings were reported by data compiled from developing countries and West Wollega, Ethiopia [27, 28]. Ten (58.82%) of the assessed facilities had laboratory commodities within the established min/max inventory. This result is better than the study conducted in Tanzania, where only 15.4% had established max/min inventor [29]. The reasons for the disagreement of the results could be due to the previous study included private health facilities and dispensaries, while the current study is done on central medical stores. However, it is worse when compared with the studies conducted in Addis Ababa [30].

The current study finding revealed wastage rate of LC was 12.94% and in value amounted to 1,186,353.3ETB (37,488.76 US$). The highest wasted LC was Acid alcohol and carbol fuchsine, which accounted for 37.5% and 31.3% of their annual budget, respectively. This finding is far above the Ethiopian hospital services transformation guidelines [31], where the wastage rate should be below 2%, and the study conducted in the West Wollega zone, Ethiopia, 8.04% [32].

However, it's better than the similar study conducted in Jimma, where the overall wastage rate was 27.2%, and some LC, such as Acid-alcohol solution and sensitivity antibiotic discs, had a wastage rate from 50 to 100% [2]. The probable reasons for the difference may be the study area and products availability level with different risks prone to expiration and damage. From the key informants, main factors that contributed to why health facilities didn't meet the recommendable wastage rate of Ethiopian hospital services transformation guideline were poor storage conditions such as insufficient storage space, nonfunctional cold chain equipment, infrequent utilization of first expire first out (FEFO) principles, lack of adequate and committed staff, infrequent supervision and feedback from the higher official. Information of products in health facilities is useful when it is accurate, complete, and timely reported for the evidence-based decision on the number of products needed, costs of its incurred, time when product required, and early preparations when emergent risks occur. On the other side with poor information recording and reporting practices the logistics judgment may be inefficient [33].

For the current study, only 280 (58.8%) bin cards were available, 90 (64.3%) and 190 (56.54%) being at hospitals and health centers, respectively. Out of available bin card, only 165 (34.66%) were updated, and 50 (35.7%), and 115 (34.2%) of them, were updated in hospitals and health centers, respectively. Overall, health facilities' data recording practices were poor, but hospitals were a bit better than health centers. This finding is comparable to the similar study conducted in.

Jimma, Southwest, Ethiopia, where 69.9% bin cards were utilized, and 57.8% were updated [2].

On the other hand, it's lower than the study conducted in SNNPRS of Ethiopia, where 81.3% and 88.8% bin cards were updated at hospitals and health centers, respectively [34]. Again lower than the finding of West Wollega, Ethiopia, where 78.4% bin cards were available and 66.9% accurate [28]. The difference might be due to data recording and reporting materials availability such as computer and manual formats, number of staff and their awareness level on data management.

Regarding data reporting, from 102 assessed RRF, only 49 % were accurate, 71% complete, and 64% timely reported to resupply facilities. The accuracy of RRF was better in hospitals (57%) than in health centers (42%). When equated to a similar study of Addis Ababa [35], it is less accurate (77.3%) but superior complete (32%) and comparative timely reporting rate. Moreover, it was less accurate at both hospitals (70.4%) and health centers (59.2%) of the study of East Gojjam zone, Ethiopia [36]. The reasons for poor data recording and reporting practices in the current study were lack of time due to workload, poor electronic information management system utilization, inadequate awareness about the importance of quality data for LCs inventory management, and lack of incentives for their extra workload.

According to the Ethiopian standard, the health facilities' acceptable storage condition criteria is 80% and above [37]. However, in the current study, only 68.2% of storage condition criteria of health facilities were achieved. Again, lower than the finding of Southern Nations, Nationalities, and People's Regional State (SNNPR) and Jimma, Ethiopia, where they fulfilled 83.3% and 70.6%, respectively [2, 34]. But, it is more than the study done at Addis Ababa, which only attained 36.36% [35]. The variation may be due to different study areas. From the key informants' interview, the factors for unacceptable storage condition criteria were lack of sufficient racks and shelves, insufficient storage space, less functional cold chain equipment, poor product zooning and arrangement, and lack of following the FEFO principles.

### 4.1 The study strength and limitation

The current study included quantitative and qualitative findings that support each other to obtain more information about inventory management of laboratory commodities. However, due to lack of complied quality data from the medical dispensary unit of health facilities, we have used the data from the main medical store to calculate wastage rate of laboratory commodities.

## 5 Conclusion

Overall, there was inadequate inventory management of laboratory commodities in current study health facilities, which was expressed with long periods of stock out, high wastage rate, unacceptable storage conditions, poor data recording, and reporting performances. Health facilities were faced many with challenges to maintain adequate inventory management, for instance, lack of sufficient and committed staff, lack of training and awareness about the importance of quality data, budget constraints, insufficient storage space, less functional cold chain equipment, frequent stock out from the supplier, short shelf life due to the nature of LCs, lack of frequent supervision and, lack of support and feedback. Therefore, EPSA and stakeholders should provide the necessary training for professionals working as store managers and laboratory managers, regularly supervise facilities to identify the strength and weaknesses of the laboratory management practices, mobilization of resources, and give attention to staff incentives.

## Declarations

### Author contribution statement

Bekele Anbase Boche; Shamoro Temam; Oliyad Kebede: Conceived and designed the experiments; Performed the experiments; Analyzed and interpreted the data; Contributed reagents, materials, analysis tools or data; Wrote the paper.

### Funding statement

This research did not receive any specific grant from funding agencies in the public, commercial, or not-for-profit sectors.

### Data availability statement

No data was used for the research described in the article.

### Declaration of interest’s statement

The authors declare no conflict of interest.

### Additional information

Supplementary content related to this article has been published online at [URL].
