# Supplementary material for: Inventory management performance for laboratory commodities and their challenges in public health facilities of Gambella Regional State, Ethiopia: A mixed cross-sectional study
Source: Heliyon. 2022 Nov 2;8(11):e11357. doi: 10.1016/j.heliyon.2022.e11357 (PMC9649967; doi:10.1016/j.heliyon.2022.e11357)
Supplement: Supplementary files [file mmc1.docx]

**Supplementary files**

**Supplementary file one:** List of laboratory commodities included in the study, Gambella regional state, January 2022

| S.N | **List of Lab Commodities** | **Unit of issue** |
| --- | --- | --- |
|  | HIV test kit 1st response | 20 test |
|  | HIV test kit 2nd response | 40test |
|  | HIV test kit 3^rd^ response | 25test |
|  | Gram stain reagent, safranin | 500ml |
|  | Gram stain reagent, iodine | 500ml |
|  | Gram stain reagent, alcohol | 500ml |
|  | Gram stain ,crystal violet | 500ml |
|  | Acid-alcohol solution | 500ml |
|  | Carbol Fuchsine 1% | Liter |
|  | Methylene blue solution | Liter |
|  | Methanol | Liter |
|  | Giemsa stain solution | Liter |
|  | Potassium hydroxide 10% | Liter |
|  | Hematology auto analyzer reagent kit | pack |
|  | Chemistry auto analyzer reagent kit, GOT (AST) | pack |
|  | Chemistry auto analyzer reagent kit, glucose | pack |
|  | Chemistry auto analyzer reagent kit, creatine | pack |
|  | Blood group /type antisera | 3*10ml |
|  | Immersion oil | 100ml |
|  | Pregnancy test kit | 40test |
|  | Urine multi-test | 150test |
|  | Hepatitis screening | 40test |
|  | RPR/VDRAL kit | 50 test |
|  | CD4 test reagents Kit | 100 |
|  | Alcohol 70% | Liter |
|  | Microscope slide | 100 |
|  | Glove | 100 |
|  | Mask | 50 |

**Supplementary file two: Measurements**

The key performance indicators adopted from the USAID delivery project logistic indicators assessment tool (LIAT) (23), and Assessment Tool for Laboratory Services (ATLAS) (24), was used to measure the inventory management performance of the health facilities.

**Accuracy of RRF** $=(\frac{stock balance on RRF reports-Ending balance on bin card}{Ending balance on bin card})*100$

**Accuracy of Bin card** $=(\frac{Ending balance on bin card-physical stock count}{physical stock count})*100$

**Percentage of fulfillment of each storage condition criteria=**

$$\left( \frac{Number of \left[ YES \right\}responses for each storage criteria}{Total number of storage facilities assessed} \right)*100$$

**Wastage rate**: It measures the value of a LC that was unusable due to damage and expiry as a percentage of total items utilized during a defined period

**Wastage rate**:$\left( \frac{value of unsable LC}{Total value of LC} \right)*100$

LC: laboratory commodity

**Supplementary file three***:* Availability and stock out of laboratory commodities in selected public health facilities of Gambella regional state, January 2022

| S.N | Name of Laboratory commodities | Available on the day of visit | | | Average frequency of SO | | | Average days out of stock | |
| --- | --- | --- | --- | --- | --- | --- | --- | --- | --- |
|  |  | Hospitals | | HC | Hospital | HC | Hospital | | HC |
| 1 | HIV test kit 1st response | 5(100%) | 12(100%) | | 0 | 0 | | 0 | 0 |
| 2 | HIV test kit 2nd response | 5(100%) | 11(91.7%) | | 0 | 1 | | 0 | 7 |
| 3 | HIV test kit 3^rd^ response | 5(100%) | 5(41.7%) | | 0 | 12 | | 0 | 104 |
| 4 | Gram stain reagent, safranin | 1(20%) | 5(41.7%) | | 5 | 7 | | 41 | 109 |
| 5 | Gram stain reagent, iodine | 4(80%) | 7(58.3%) | | 1 | 2 | | 30 | 20 |
| 6 | Gram stain reagent, alcohol | 5(100%) | 7(58.3%) | | 0 | 2 | | 0 | 24 |
| 7 | Gram stain reagent, crystal violet | 1(20%) | 3(25%) | | 2 | 10 | | 39 | 66 |
| 8 | Acid-alcohol solution | 4(80%) | 10(83.3%) | | 1 | 2 | | 15 | 51 |
| 9 | Carbol Fuchsine 1% | 5(100%) | 9(75%) | | 1 | 2 | | 44 | 9 |
| 10 | Methylene blue solution | 4(80%) | 10(83.3%) | | 1 | 2 | | 3 | 21 |
| 11 | Methanol | 4(80%) | 7(58.3%) | | 2 | 6 | | 20 | 49 |
| 12 | Giemsa stain solution | 4(80%) | 7(58.3%) | | 1 | 4 | | 13 | 67 |
| 13 | Potassium hydroxide 10% | 1(20%) | 2(16.7%) | | 5 | 9 | | 54 | 118 |
| 14 | Hematology auto analyzer reagent kit | 0(0%) | 0(0%) | | 7 | 1 | | 51 | 180 |
| 15 | Chemistry auto analyzer reagent kit, GOT (AST) | 2(40%) | 0(0%) | | 1 | 1 | | 15 | 360 |
| 16 | Chemistry auto analyzer reagent kit, glucose | 2(40%) | 0(0%) | | 1 | 1 | | 15 | 360 |
| 17 | Chemistry auto analyzer reagent kit, creatine | 2(40%) | 0(0%) | | 1 | 1 | | 15 | 360 |
| 18 | Blood group /type antisera | 2(40%) | 6(50%) | | 1 | 10 | | 31 | 107 |
| 19 | Immersion oil | 4(80%) | 11(91.7%) | | 1 | 1 | | 7 | 26 |
| 20 | Pregnancy test kit | 4(80%) | 9(75%) | | 1 | 5 | | 16 | 77 |
| 21 | Urine multi-test | 3(60%) | 6(50 %\|) | | 4 | 2 | | 36 | 31 |
| 22 | Hepatitis screening | 4(80%) | 9(75%) | | 1 | 1 | | 12 | 23 |
| 23 | RPR/VDRAL kit | 2(40%) | 7(58.3%) | | 5 | 2 | | 50 | 26 |
| 24 | CD4 test reagents Kit | 2(40%) | 1(8.3%) | | 4 | 23 | | 27 | 163 |
| 25 | Alcohol 70% | 4(80%) | 11(91.7%) | | 1 | 1 | | 5 | 9 |
| 26 | Microscope slide | 4(80%) | 11(91.7%) | | 1 | 1 | | 10 | 12 |
| 27 | Glove | 4(80%) | 8(66.7%) | | 1 | 2 | | 7 | 22 |
| 28 | Mask | 5(100%) | 11(91.7%) | | 0 | 1 | | 0 | 7 |
| Overall average | | **65.71%** | **55.06%** | | **1.8** | **3.8** | | **19.86** | **96.32** |

HC= health center, LCs= laboratory commodities, SO=stock out

**Supplementary file four**: Percentage of health facilities that fulfilled acceptable Storage criteria in Gambella regional state, January 2022

| S. No | Storage practice | Yes (%) | No (%) |
| --- | --- | --- | --- |
| 1 | Are products arranged with clear identification? | 13(76.5%) | 4(23.5%) |
| 2 | Are products arranged with accessible to FEFO? | 12(70.6%) | 5(29.4%) |
| 3 | Are cartons and products in good condition? | 12 (70.6%) | 5 (29.4%) |
| 4 | Does facility separate damaged & expired LCs? | 3(17.6%) | 14(82.4%) |
| 5 | Are products protected from direct sun light? | 16(94.1%) | 1(5.9%) |
| 6 | Are cartons and products protects from water and humidity? | 15(88.2%) | 2(11.8%) |
| 7 | Does the storage area free from harmful insects and rodents? | 13(76.5%) | 4(23.5) |
| 8 | Does the storage area secured & access limited? | 17(100%) | 0 (0%) |
| 9 | Are products stored at appropriate temperature? | 5(29.4%) | 12(70.6%) |
| 10 | Are roofs maintained in good conditions? | 15 (88.2%) | 2(11.8%) |
| 11 | Are store room maintained in good condition? | 16 (94.1%) | 1(5.9%) |
| 12 | Does the current space sufficient? | 3 (17.6%) | 14(82.4%) |
| 13 | Is fire safety equipment available & accessible? | 10 (58.8%) | 7(41.2%) |
| 14 | Does diagnostic products stored separately from chemicals? | 15(88.2%) | 2(11.8%) |
| 15 | Are products are stacked 10cm off the floor? | 6(35.3%) | 11(64.7%) |
| 16 | Products stacked 30cm away from the wall | 14(82.4%) | 3(17.6%) |
| 17 | Products are stacked no more than 2.5m high | 12(70.6%) | 5(29.4%) |
|  | Overall average | 68.2% | 31.8% |

FEFO= first expired first out, LCs= laboratory commodities
